# Pontibacter rufus sp. nov., Pontibacter humidus sp. nov. and Pontibacter coccineus sp. nov. isolated from UV-irradiated soil in Korea

**DOI:** 10.1099/ijsem.0.006755

**Published:** 2025-04-28

**Authors:** Seona Park, Hyang Burm Lee, Sathiyaraj Srinivasan, Myung Kyum Kim

**Affiliations:** 1 Department of Bio & Environmental Technology, College of Natural Science, Seoul Women’s University, Seoul 01797, South Korea; 2 Environmental Microbiology Lab, Department of Agricultural Biological Chemistry, College of Agriculture & Life Sciences, Chonnam National University, Gwangju 61186, South Korea

**Keywords:** 16S rRNA, *Hymenobacteraceae*, *Pontibacter*, taxonomy, whole genome

## Abstract

Three novel bacterial strains, 172403-2^T^, BT310^T^ and BT731^T^, were isolated from UV-irradiated soil samples collected in South Korea. All strains are Gram-negative, aerobic, non-motile and grow at 20–35 °C, optimally at 30 °C and pH 7.0. The optimal NaCl concentration for strains 172403-2^T^ and BT310^T^ is 1.5%, while strain BT731^T^ grows optimally in the absence of NaCl (0.0%). Phylogenetic analysis based on 16S rRNA gene sequences positioned these strains within the genus *Pontibacter*, with strain 172403-2^T^ closest to *Pontibacter chitinilyticus* (95.96%), BT310^T^ to *Pontibacter pudoricolor* (97.87%) and BT731^T^ to *Pontibacter virosus* (98.06%). Cellular fatty acid profiles identified C_15:0_ iso and Summed Feature 4 as predominant fatty acids. All strains contained ubiquinone MK-7 and phosphatidylethanolamine as major respiratory quinone and primary polar lipids, respectively. Genome sizes were 5.08 Mb for 172403-2^T^, 4.29 Mb for BT310^T^ and 4.66 Mb for BT731^T^, with average nucleotide identity and digital DNA–DNA hybridization values with other *Pontibacter* species ranging between 70.41%–88.56% and 11.55%–24.66%, respectively. These biochemical, chemotaxonomic and phylogenetic analyses confirm that strains 172403-2^T^, BT310^T^ and BT731^T^ represent three novel species of *Pontibacter*, proposed as *Pontibacter rufus* sp. nov. (172403–2^T^ = KCTC 62072 ^T^=NBRC 114967^T^), *Pontibacter humidus* sp. nov. (BT310^T^ = KCTC 72363^T^ = NBRC 114846^T^) and *Pontibacter coccineus* sp. nov. (BT731^T^ = KCTC 92910^T^ = NBRC 116069^T^).

## Introduction

*Pontibacter* is an established genus within the class *Cytophagia*, belonging to the family *Hymenobacteraceae*. This family includes eight genera, as documented in the List of Prokaryotic Names with Standing in Nomenclature [1]. *Pontibacter* was initially defined in 2005, who designated *Pontibacter actiniarum* as the type species [2]. As of May 2024, the genus *Pontibacter* had expanded to comprise 46 validly published species [1], which have been discovered in a wide range of environmental settings. These environments include soil [3,4], saline–alkaline soils [5] and mangrove sediments [6]. Additionally, they have been found in rhizosphere soils [7,8], marine habitats [9] and extreme or high-stress environments, such as radiation-affected areas [10] and deserts [11,13].

The cells of the genus *Pontibacter* exhibit Gram-negative characteristics, are capable of aerobic respiration, and have a rod-shaped morphology. They can be either motile or non-motile and typically form red or pink colonies. Additionally, the typical chemotaxonomic characteristics of the *Pontibacter* genus include phosphatidylethanolamine (PE), aminophospholipid (APL) and several unidentified phospholipids (PL) as the predominant polar lipids; MK-7 as the major respiratory quinone; and iso-C_15:0_, C1_6:1_ *ω5*c, Summed Feature 3 (C_16:1_ *ω7*c/C1_6:1_ *ω6*c) and Summed Feature 4 (C_17:1_ anteiso B/C_17:1_ iso I) as the primary cellular fatty acids [14].

Several *Pontibacter* species, including *Pontibacter pudoricolor* BT214^T^, *Pontibacter russatus* BT326^T^ [10], *Pontibacter korlensis* X14-1^T^ [15] and *Pontibacter aquaedesilientis* JH31^T^ [16], demonstrate resistance to UV radiation. These species contain genes associated with DNA repair, including UV resistance genes, such as the *rec* family (*recA*, *recR*, *recO*, *recN*, *recQ*), and the UvrABC proteins, which improve their ability to endure environmental stresses like desiccation, high salinity and radiation [17]. The presence of these genes indicates a robust mechanism for coping with harsh conditions, highlighting their potential for biotechnological applications

To investigate microbial diversity, we gathered soil from UV-irradiated soil samples from Pyeongchang-gun, Uijeongbu-si and Namyangju-si in South Korea. Our study identified three novel Gram-negative bacterial strains, 172403-2^T^, BT310^T^ and BT731^T^, all belonging to the genus *Pontibacter*. This study comprehensively characterizes these novel strains through a polyphasic approach.

## Methods

### Organism and culture conditions

Strains 172403-2^T^, BT310^T^ and BT731^T^ were isolated from UV-irradiated soil samples collected in Pyeongchang-gun (37° 43′ 35.8′ N 128° 37′ 57.1′ E), Uijeongbu-si (37° 45′ 34.3′ N 127° 04′ 43.0′ E) and Namyangju-si (37° 35′ 11.2″ N 127° 13′ 15.2″ E), Republic of Korea. Each soil sample was exposed to UV radiation at a wavelength of 254 nm using a CX-2000 UV Crosslinker (UVP, USA) at a total dose of 900 J, simulating high-radiation environmental conditions as previously described [18,19]. The UV-irradiated soil samples were collected in sterile tubes and immediately transferred to the laboratory for microbial isolation. For the isolation process, 1 g of each soil sample was suspended in 10 ml of sterile normal saline (1/10 dilution) and incubated at 37 °C for 1 h. The suspension was then serially diluted, and a 100 µl aliquot of each dilution was spread onto Reasoner’s 2A (R2A, BD Difco) agar plates and incubated at 25 °C. After 3 days of incubation, several colonies were observed and selected for purification. The 16S rRNA gene sequences of the isolated strains were compared and analysed using the EzBioCloud server [20], and based on their low 16S rRNA gene similarity, 172403-2^T^, BT310^T^ and BT731^T^ strains were selected for polyphasic analysis. The purified strains were preserved in R2A broth containing 20% (v/v) glycerol at −80 °C.

### Morphological, physiological and biochemical analysis

The cell morphologies of strains 172403-2^T^, BT301^T^ and BT731^T^ were observed using transmission electron microscopy (JEOL, JEM1010) after a 3-day incubation on R2A agar plates at 30 °C. Motility was assessed on R2A agar plates containing 0.2% agar, and the Gram reaction was performed using a standard Gram reaction kit from bioMérieux. Growth on different culture media was observed using R2A agar, nutrient agar (NA, BD Difco), tryptic soy agar (TSA, BD Difco), MacConkey agar (MCA, BD Difco) and lysogeny broth (LB, BD Difco). Additionally, growth at various temperatures (4, 10, 15, 20, 25, 30, 35, 37 and 40 °C) and pH levels (pH 5.0–9.0 in 0.5 unit increments) was examined on R2A broth, and pH-dependent growth was further analysed with acetate buffer (pH 5.0–6.5) and phosphate buffer (pH 7.0–9.0) at a final concentration of 100 mM. Sodium chloride (NaCl) tolerance was tested across concentrations ranging from 0.5% to 7.0% (w/v) at 1.0% intervals. Oxidase activity was determined using 1% (w/v) tetramethyl-*p*-phenylenediamine [21], while catalase activity was evaluated by observing bubble production upon application of 3% (v/v) hydrogen peroxide solution [22]. Carbon source utilization and fermentation were assessed with the API 20NE test kit (bioMérieux), and enzymatic activities were measured using the API ZYM test kit (bioMérieux), both according to the manufacturer’s instructions.

### Phylogenetic analysis and genome sequencing

The genomic DNA of strains 172403-2^T^, BT310^T^ and BT731^T^ was extracted using a Qiagen DNA extraction kit. The 16S rRNA gene was amplified via standard polymerase chain reaction (PCR) using the bacterial primer pair 27F and 1492R [23]. The purified PCR products were sequenced by Bionics, Republic of Korea. The taxonomic classification of the strains was determined by comparing their 16S rRNA gene sequences with those in the EzBioCloud server, which provides comprehensive and accurate assessments. Additionally, the National Center for Biotechnology Information (NCBI) Basic Local Alignment Search Tool (blast) was used to collect updated information. Closely related type strains were downloaded from GenBank [24], and multiple alignments of the 16S rRNA gene sequences were performed using the EzEditor2 program [20]. From the aligned sequences, phylogenetic trees were constructed using mega11 software [25] using three algorithms: neighbour-joining (NJ) [26], maximum likelihood (ML) [27] and maximum parsimony (MP) [28]. Bootstrap analyses with 1000 resamples were performed to evaluate tree reliability [27]. To support the phylogenetic analysis, evolutionary distances were calculated using the Kimura two-parameter model [29].

For genome sequencing, the genomic DNA from strains 172403-2^T^, BT310^T^ and BT731^T^ was extracted using a Solgent genomic DNA extraction kit according to the manufacturer’s instructions. After extraction, DNA concentrations were measured, and sequencing libraries were prepared using the Nextera DNA Flex Library Prep Kit from Illumina. Whole-genome sequencing was performed on the iSeq 100 platform using the 2×150 bp paired-end reads run configuration. The sequencing data were assembled using SPAdes 3.13.0, a software developed by the Algorithmic Biology Lab at St. Petersburg Academic University, Russian Academy of Sciences.

The genome sequences of strains 172403-2^T^, BT310^T^ and BT731^T^ were submitted to the GenBank database and annotated using the Prokaryotic Genome Annotation Pipeline (PGAP) from the NCBI. Average nucleotide identity (ANI) values were calculated using the EzBioCloud web tool [30]. Digital DNA–DNA hybridization (dDDH) values were determined using the Genome-to-Genome Distance Calculator (GGDC) tool, using ‘formula 2’ for enhanced precision [31]. For taxonomic classification, the Genome Taxonomy Database Toolkit (GTDB-Tk) was utilized to generate a GTDB tree, providing a taxonomy estimation for the novel strains. This involved a concatenated multiple sequence alignment of 120 marker genes, as specified in GTDB-Tk version 2.3.0 [32]. The resulting phylogenetic tree was visualized using the Interactive Tree Of Life (iTOL) software [33].

Whole genome assemblies were gathered for type strains closely related to the novel strains, as identified in the GTDB tree visualized with iTOL, and a whole-genome-based phylogenetic tree was constructed using these assemblies based on the Up-to-date Bacterial Core Gene (UBCG) set pipeline. This method utilizes a concatenated sequence dataset of 92 single-copy bacterial core genes for phylogenetic reconstruction [34]. The functional genes were also analysed and categorized to assess these strains' metabolic characteristics and ecological roles using the Rapid Annotation using Subsystem Technology (RAST) server [35,36].

### UV radiation resistance

The cells were exposed to radiation at a wavelength of 254 nm using a CX-2000 UV Crosslinker (UVP, USA) with different dose modifications, as previously described [18,19]. Strains 172403-2^T^, BT310^T^ and BT731^T^ were subjected to UV radiation, and their survival ratios were assessed on R2A agar plates (Difco) using cells in the early stationary phase (≈ 10^9^ c.f.u. ml^−1^). In the UV resistance test, *Escherichia coli* K-12 (= KCTC 1116) was utilized as the negative control, and *Deinococcus radiodurans* R1^T^ (= DSM 20539^T^ = NBRC 15346ᵀ) as the positive control. The strains' colony-forming units (CFU) were determined, and the survival ratio was assessed using this information.

### Chemotaxonomic characteristics

The polar lipids of strains 172403-2^T^, BT310^T^ and BT731^T^ were extracted and analysed using two-dimensional thin-layer chromatography (TLC), following the established method [37]. The separated polar lipids were identified using specific reagents: chloroform, methanol and water mixed in ratios of 9:10:3 v/v/v for the first dimension and 5:10:4 v/v/v for the second dimension [38]. The TLC plate was treated with ethanolic molybdatophosphoric acid, *α*-naphthol in sulfuric acid reagent, Dragendorff’s reagent, a ninhydrin spray solution and molybdenum blue reagent to identify total lipids, glycolipids, phosphatidylcholine, amino groups and phosphorus-containing lipids, respectively. Quinones from each strain were extracted using Sep-Pak Vac cartridges (Waters) and analysed by high-performance liquid chromatography (HPLC) [39]. Cellular fatty acids were analysed by culturing on R2A agar for 3 days at 28 °C, followed by saponification, methylation and extraction [40]. The resulting fatty acid methyl esters (FAME) were identified using the Sherlock Microbial Identification System V6.01 (MIS, database TSBA6, MIDI Inc., Newark, DE, USA).

## Results and discussion

### Morphology, physiology and biochemical characteristics

**Fig. 1.**
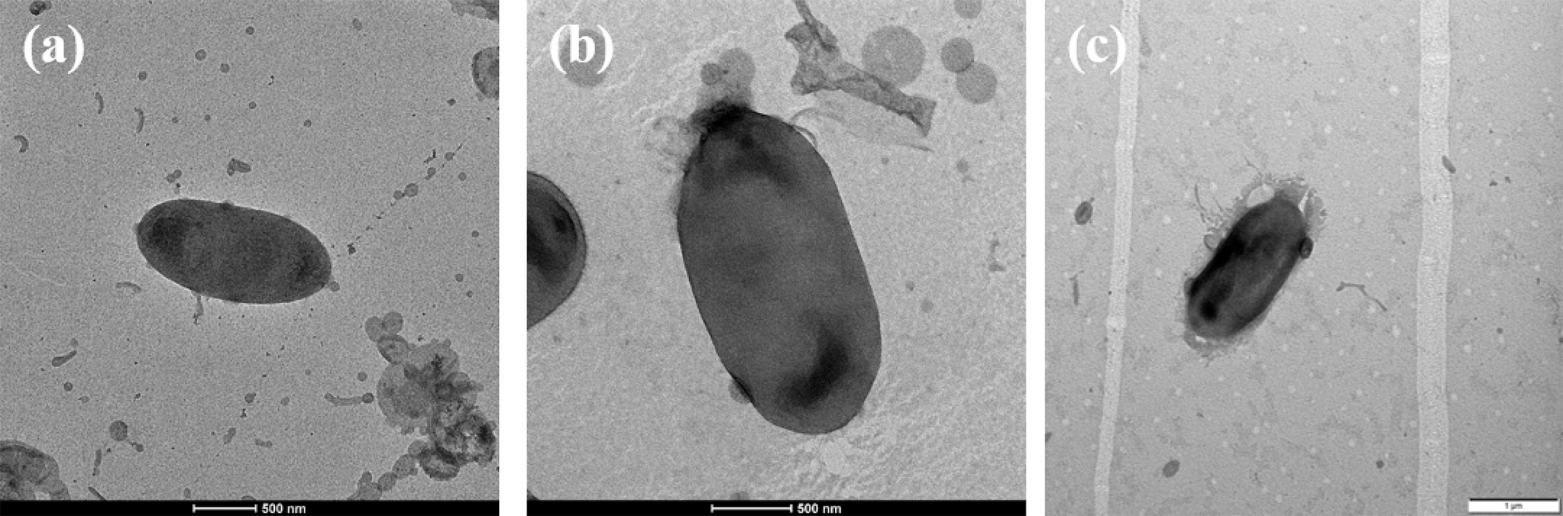
Transmission electron micrographs of strains 172403-2^T^ (**a**), BT310^T^ (**b**) and BT731^T^ (**c**).

The strains 172403-2^T^, BT310^T^ and BT731^T^ were obtained from UV-irradiated soil samples collected in Pyeongchang-gun, Uijeongbu-si and Namyangju-si, Republic of Korea. All three strains were Gram-stain negative, non-flagellated and rod shaped (Fig. 1). After 72 h of incubation at 30 °C on R2A agar, distinctive colony morphologies were observed for each strain: strain 172403-2^T^ exhibited red, circular, convex and mucoid colonies; strain BT310^T^ displayed light pink, circular, convex and moist colonies; and strain BT731^T^ showed orange-red, circular, convex and smooth colonies. The growth of the strains was evaluated across different temperature ranges and NaCl concentrations. Specifically, strains 172403-2^T^, BT310^T^ and BT731^T^ showed growth at 10–37, 20–37 and 10–35 °C, respectively, in the absence of NaCl. Moreover, strains 172403-2^T^ and BT310^T^ exhibited growth in NaCl concentrations ranging from 0.0% to 4.0%, whereas strain BT731^T^ grew within NaCl concentrations ranging from 0.5% to 3.5%. Additionally, all three strains grew well at pH levels of 6.0–8.0.

All three strains exhibited positive oxidase activity, but BT310^T^ stood out from 172403-2^T^ and BT731^T^ by demonstrating negative catalase activity. In the API 20NE test, strain 172403-2^T^ showcased positive reactions for *β*-galactosidase (PNPG) and protease, while BT731^T^ showed positive results for urease and d-mannose. In the API ZYM test, strain 172403-2^T^ exhibited positive activity for naphtol-AS-BI-phosphohydrolase and *N*-acetyl-*β*-glucosaminidase. Conversely, strain BT310^T^ demonstrated positive reactions for esterase (C4), esterase (C8), leucine arylamidase, valine arylamidase and trypsin. Strain BT731^T^, on the other hand, presented positive reactions for lipase (C14), leucine arylamidase, cystine arylamidase, *α*-chymotrypsin, acid phosphatase, naphtol-AS-BI-phosphohydrolase and *β*-glucuronidase. These observed differences clearly distinguish them as three distinct novel species, with detailed API 20NE and API ZYM results provided as follows.

In the API 20NE test, strain 172403-2^T^ displays positive reactions for *β*-glucosidase (aesculin hydrolysis), protease (gelatin hydrolysis) and *β*-galactosidase (PNPG), but negative reactions for nitrate reduction, indole production, glucose fermentation, arginine dihydrolase, urease and for all tested assimilation substrates, including d-glucose, l-arabinose, d-mannitol, d-maltose, potassium gluconate, d-mannose, *N*-acetyl-d-glucosamine, caprate, adipate, l-malate, citrate and phenylacetate. Strain BT310^T^ shows weak positive results for *β*-glucosidase (aesculin hydrolysis) but negative results for nitrate reduction, indole production, glucose fermentation, arginine dihydrolase, urease, protease (gelatin hydrolysis), *β*-galactosidase (PNPG) and for all tested assimilation substrates. Strain BT731^T^ shows positive results for urease, *β*-glucosidase (aesculin hydrolysis) and assimilation of d-mannose but negative results for nitrate reduction, indole production, glucose fermentation, arginine dihydrolase, protease (gelatin hydrolysis), *β*-galactosidase (PNPG) and all other tested assimilation substrates.

In the API ZYM test, strain 172403-2^T^ produces positive reactions for alkaline phosphatase, leucine arylamidase, valine arylamidase, acid phosphatase, naphtol-AS-BI-phosphohydrolase and *N*-acetyl-*β*-glucosaminidase. Weakly positive reactions were observed for esterase (C4), esterase (C8), cystine arylamidase and *α*-galactosidase, and negative reactions for lipase (C14), trypsin, *α*-chymotrypsin, *β*-galactosidase (ONPG), *β*-glucuronidase, *α*-glucosidase (starch hydrolysis), *β*-glucosidase, *α*-mannosidase and *α*-fucosidase. Strain BT310^T^ shows positive reactions for alkaline phosphatase, esterase (C4), esterase (C8), leucine arylamidase, valine arylamidase and trypsin and negative results for lipase (C14), cystine arylamidase, *α*-chymotrypsin, acid phosphatase, naphtol-AS-BI-phosphohydrolase, *α*-galactosidase, *β*-galactosidase (ONPG), *β*-glucuronidase, *α*-glucosidase (starch hydrolysis), *β*-glucosidase, *N*-acetyl-*β*-glucosaminidase, *α*-mannosidase and *α*-fucosidase. Strain BT731^T^ displayed positive activity for lipase (C14), leucine arylamidase, cystine arylamidase, *α*-chymotrypsin, acid phosphatase, naphtol-AS-BI-phosphohydrolase and *β*-glucuronidase. Weakly positive activity was observed for alkaline phosphatase, esterase (C4), valine arylamidase, trypsin and *N*-acetyl-*β*-glucosaminidase, whereas negative activity was recorded for esterase (C8), *α*-galactosidase, *β*-galactosidase (ONPG), *α*-glucosidase (starch hydrolysis), *β*-glucosidase, *α*-mannosidase and *α*-fucosidase.

Furthermore, strains 172403-2^T^, BT310^T^ and BT731^T^ exhibited distinct enzymatic and biochemical characteristics compared to their closely related species. For example, 172403-2^T^ was negative for *α*-glucosidase activity in the enzyme activity test and did not assimilate l-arabinose in the assimilation test, whereas its closely related species were positive or weak positive for both. BT310^T^ exhibited negative results for cystine arylamidase, *α*-chymotrypsin, naphtol-AS-BI-phosphohydrolase, *N*-acetyl-*β*-glucosaminidase and protease activity, contrasting with positive or weak positive results in its closely related species. Strain BT731^T^ showed negative results for esterase (C8) and acid phosphatase but was positive for lipase (C14), a characteristic absent in related species. It was also negative for protease activity, contrasting with consistently positive reactions in related species. From the observed differences in fermentation, assimilation properties and enzymatic activities, these three strains can be clearly differentiated from their closest neighbours within the *Pontibacter* genus, supporting their classification as distinct species [41]. These findings strongly suggest that the unique characteristics of strains 172403-2^T^, BT310^T^ and BT731^T^ justify their classification as novel species. Detailed characteristics are summarized in Tables1  2.

**Table 1.** Characteristics of strain 172403-2^T^ and closely related species (+, positive; −, negative; w, weak positive). Numbers denote the strains: 1, 172403-2^T^; 2, *Pontibacter chitinilyticus* 17 gy-14^T^; 3, *Pontibacter arcticus* 2b14^T^; and 4, *Pontibacter liquoris* NBU2971^T^. The data for strain 172403-2^T^ and the reference strains were obtained in this study unless otherwise indicated

Characteristic	1	2	3	4
Length (μm)	2.0–2.7	1.0–1.7	1.3–2.1	1.1–1.3
Width (μm)	1.0–1.5	0.6–0.8	0.6–1.0	0.7–0.8
Gliding motility	−	−	+	−
Colony colour	Red	Orange red	Red	Pink
Oxidase	+	+	−	+
Optimum growth temperature (°C)	30	28–30	28	28
Temperature range (°C)	10–40	7–37	4–37	4–37
NaCl % range	0.0–4.0	0.0–5.5	0.0–2.5	0.0–4.0
**API ZYM**				
Trypsin	−	−	+	+
*α*-galactosidase	w	−	−	+
*β*-galactosidase (ONPG)	−	+	−	+
*α*-glucosidase (starch hydrolysis)	−	+	+	+
*β*-glucosidase	−	+	−	+
*N-*acetyl-*β*-glucosaminidase	+	+	−	+
*α*-mannosidase	−	w	−	+
**API 20NE**				
Arginine dihydrolase	−	−	+	−
*β*-glucosidase	+	+	−	+
Protease	+	−	+	−
*β*-galactosidase (PNPG)	+	+	−	+
d-glucose	−	+	−	−
l-arabinose	−	w	+	+
d-mannose	−	−	+	+
d-maltose	−	−	+	+
G+C content (mol%)	48.6	50.5 [3]	45.5 [8]	51.3 [51]

All strains had positive results for alkaline phosphatase, leucine arylamidase, valine arylamidase and naphtol-AS-BI-phosphohydrolase and negative results for *α*-chymotrypsin, nitrate reduction, production of indole and urease.

**Table 2.** Characteristics of strains BT310^T^ and BT731^T^ and closely related species (+, positive; −, negative; w, weak positive). Numbers denote the strains: 1, BT310^T^; 2, BT731^T^; 3, *Pontibacter pudoricolor* BT214^T^; 4, *Pontibacter populi* HYL7-15^T^; 5, *Pontibacter virosus* W14^T^; and 6, *Pontibacter amylolyticus* 9-2^T^. The data for strains BT310^T^, BT731^T^ and the reference strains were obtained in this study unless otherwise indicated

Characteristic	1	2	3	4	5	6
Length (μm)	2.2–2.4	1.8–2.6	1.0–1.4	1.0–2.2	1.0–1.6	1.0–1.2
Width (μm)	0.9–1.1	0.7–1.2	0.4–0.6	0.4–0.6	0.4–0.6	0.6–1.0
Gliding motility	−	−	−	−	+	−
Colony colour	Light pink	Orange red	Red	Pink	Red	Red
Catalase	+	−	+	+	+	+
Optimum growth temperature (°C)	30	30	25	30	28	35
Temperature range (°C)	20–37	10–35	10–37	4–37	20–40	4–40
NaCl % range	0.0–4.0	0.0–3.5	0.0–4.0	0.0–4.0	0.0–5.0	0.0–5.0
**API ZYM**						
Esterase (C8)	+	−	+	w	w	+
Lipase (C14)	−	+	−	−	−	−
Cystine arylamidase	−	+	+	w	w	+
*α*-chymotrypsin	−	+	+	w	w	+
Acid phosphatase	−	+	−	−	w	+
Naphtol-AS-BI-phosphohydrolase	−	+	w	w	w	+
*β*-glucuronidase	−	+	−	−	−	+
*α*-glucosidase (starch hydrolysis)	−	−	−	w	−	+
*N*-acetyl-*β*-glucosaminidase	−	w	w	w	+	+
**API 20NE**						
Urease	−	+	−	−	−	+
*β*-glucosidase	w	+	w	−	w	−
Protease	−	−	w	+	+	+
d-mannose	−	+	−	−	−	−
Citrate	−	−	−	−	−	+
G+C content (mol%)	45.2	51.3	45.4	45 [52]	50.1 [53]	51.8 [54]

All strains had positive results for leucine arylamidase and negative results for *α*-galactosidase, *β*-galactosidase (ONPG), *β*-glucosidase, *α*-mannosidase, *α*-fucosidase, nitrate reduction, production of indole, production of acid from glucose, arginine dihydrolase, *β*-galactosidase (PNPG), d-glucose, l-arabinose, d-mannitol, *N*-acetyl-d-glucosamine, d-maltose, potassium gluconate, caprate, adipate, l-malate, citrate and phenylacetate.

### Phylogenetic analysis

**Fig. 2.**
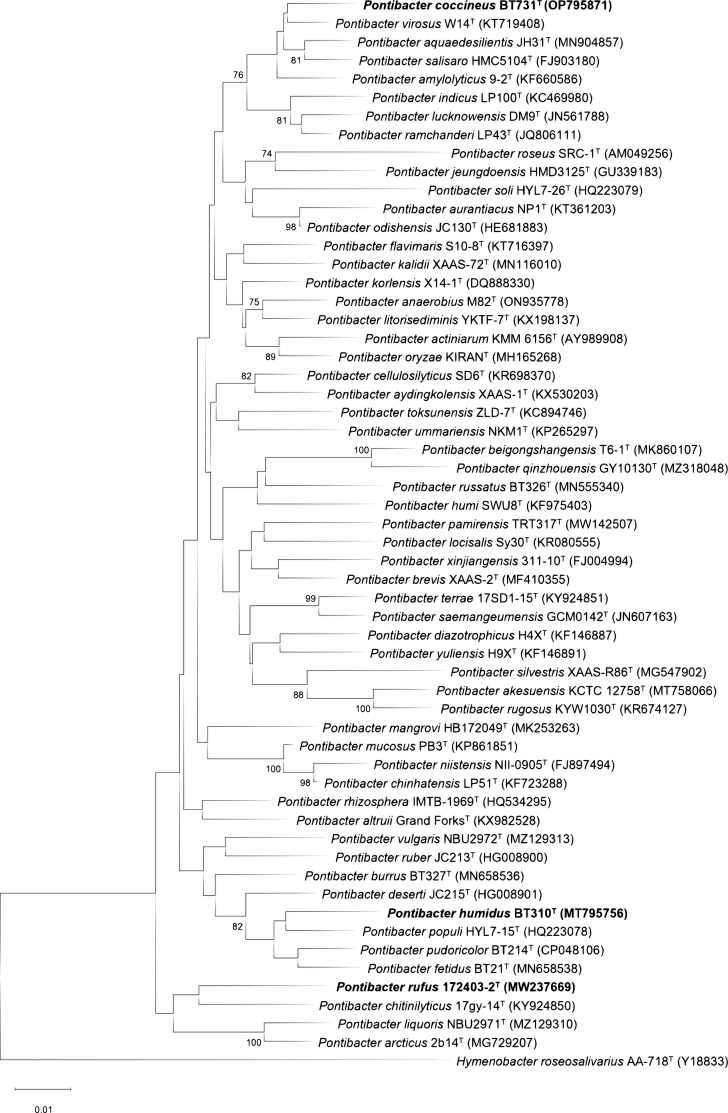
Neighbour-joining phylogenetic tree based on 16S rRNA gene sequences showing these positions of strains 172403-2^T^, BT310^T^ and BT731T^T^ among other species of the genus *Pontibacter*. The numbers at the nodes are bootstrap percentages (> 70%). *Hymenobacter roseosalivarius* AA-718^T^ was used as the outgroup. The bar represents 0.01 substitutions per nucleotide position.

Based on 16S rRNA gene sequence similarity, strains 172403-2^T^, BT310^T^ and BT731^T^ were assigned to the family *Hymenobacteraceae*, displaying notable sequence similarities to those of species belonging to the genus *Pontibacter*. Phylogenetic analyses using NJ, ML and MP methods provided further detail. Strain 172403-2^T^ showed the highest 16S rRNA gene sequence similarity, 95.96%, with *Pontibacter chitinilyticus* 17 gy-14^T^ but was phylogenetically closest to *Pontibacter arcticus* 2b14^T^ and *Pontibacter liquoris* NBU2971^T^. Strain BT310^T^ showed the highest sequence similarity, 97.87%, with *Pontibacter pudoricolor* BT214^T^ but was closest phylogenetically to *Pontibacter populi* HYL7-15^T^. Although *Pontibacter pudoricolor* BT214^T^ has been described as a closely related strain to BT310^T^, it is currently considered effectively but not validly published. Strain BT731^T^ was consistently closest, both in sequence similarity, 98.06%, and phylogenetically, to *Pontibacter virosus* W14^T^. Although the NJ algorithm (Fig. 2) provided an initial placement of the strains within distinct clusters in the genus *Pontibacter*, the ML method (Fig. S1, available in the online Supplementary Material) was more accurate. The ML analysis accounts for varying evolutionary rates across sites, offering a statistically stronger estimate of evolutionary relationships. This improved accuracy reinforces the classification of these strains as distinct species [42]. The MP analysis (Fig. S2) further supported these phylogenetic relationships.

Additionally, in the reconstructed phylogenetic analysis using the genome-based UBCG (Up-to-date Bacterial Core Gene) tool, strain 172403-2^T^ was closely aligned with *Pontibacter chitinilyticus* 17 gy-14^T^, while strain BT310^T^ was most closely related to *Pontibacter pudoricolor* BT214^T^. Similarly, strain BT731^T^ was positioned near *Pontibacter amylolyticus* CGMCC 1.12749^T^ in the UBCG phylogenetic tree (Fig. S3). Despite these close relationships, the phylogenetic positioning of strains 172403-2^T^, BT310^T^ and BT731^T^ was sufficiently distinct to propose their classification as novel species within the genus *Pontibacter*. These genomic and phylogenetic results reinforce the proposal to classify strains 172403-2^T^, BT310^T^ and BT731^T^ as new species in the *Pontibacter* genus.

### Whole genome similarities

Genomic sequencing revealed that strain 172403-2^T^ had a genome size of 5 076 851 bp with a sequencing coverage of 29.70X. The genome assembly comprised 41 contigs, with an N50 size of 363 827 bp and an L50 of 5, containing 4951 coding sequences (CDSs), 38 tRNA genes and a DNA G+C content of 48.6% (GenBank accession: JADQDR000000000). Strain BT310^T^ exhibited a genome size of 4 294 440 bp, with a coverage of 45.80×, 9 contigs, an N50 size of 1 220 683 bp and an L50 of 2. This genome contained 3901 CDSs, 38 tRNA genes and a DNA G+C content of 45.2% (GenBank accession: JAELXU000000000). Similarly, strain BT731^T^ had a genome size of 4 655 665 bp, a coverage of 38.34× and 12 contigs, with an N50 size of 627 395 bp and an L50 of 4. The genome comprised 4347 CDSs, 41 tRNA genes and a DNA G+C content of 51.3% (GenBank accession: JAUOTN000000000). For comparative analysis, genome sequences of closely related species were retrieved from GenBank. Detailed assembly statistics, including contigs, N50 size, L50, genome size, coverage, CDSs, tRNA genes and G+C content for the three novel strains and reference species, are summarized in Table S1.

The ANI values between strain 172403-2^T^ and *P. chitinilyticus*, *P. liquoris* and *P. arcticus* were 76.37%, 76.87% and 73.05%, respectively. Strain BT310^T^ had an ANI value of 88.56% when compared to *P. pudoricolor* and 83.46% when compared to *P. populi*, and strain BT731^T^ had an ANI value of 85.45% when compared to *P. virosus*. The dDDH values between strain 172403-2^T^ and *P. chitinilyticus*, *P. liquoris* and *P. arcticus* were 20.62%, 19.93% and 22.51%, respectively. Strain BT310^T^ showed dDDH values of 11.55% and 15.82% when compared to *P. pudoricolor* and *P. populi*, respectively, and strain BT731^T^ showed a dDDH value of 14.14% when compared to *P. virosus*. The ANI and dDDH values of all three novel strains were significantly below the threshold for prokaryotic species delineation [43]. These ANI and dDDH results confirm the phylogenetic distinctions observed in the UBCG tree, and the ANI and dDDH values between strains 172403-2^T^, BT310^T^ and BT731^T^, along with closely related species, are summarized in Tables S2–S4.

### Functional genome annotation

The functional genome annotation of the novel strains was performed using the RAST server [35]. The analysis identified 264 subsystems in strain 172403-2^T^, 255 in strain BT310^T^ and 260 in strain BT731^T^, highlighting their diverse functional capabilities (Figs S4–S6). These results underscore the metabolic diversity and ecological potential of the strains.

Subsystem distribution analysis revealed that amino acids and derivatives accounted for the largest proportion in all strains, comprising 19.62% in strain 172403-2^T^, 16.89% in strain BT310^T^ and 20.44% in strain BT731^T^. In strain 172403-2^T^, this was followed by carbohydrates (14.88%) and cofactors, vitamins, prosthetic groups, pigments (12.30%). In strain BT310^T^, cofactors, vitamins, prosthetic groups, pigments (13.12%) and protein metabolism (11.05%) accounted for the most considerable proportions. For strain BT731^T^, protein metabolism (11.66%) and cofactors, vitamins, prosthetic groups, pigments (11.33%) followed. These distributions suggest functional specialization and adaptation to diverse ecological niches.

Subsystems unique to each strain emphasize their specific metabolic capabilities. Strain 172403-2^T^ harboured terminal cytochrome C oxidases and proline synthesis subsystems, supporting oxidative stress management and osmoregulation [44,45]. Strain BT310^T^ featured glycerolipid and glycerophospholipid metabolism, crucial for membrane integrity and environmental stress response [46]. Strain BT731^T^ contained the urea decomposition subsystem, facilitating nitrogen cycling and acid resistance [47]. These annotations highlight the strains' genomic stability and ability to adapt to diverse environmental conditions.

In addition to subsystem annotation, DNA repair-related proteins were analysed using the BLASTp algorithm and RAST’s function-based comparison feature. Key proteins, including those from the UvrABC system and RecA, were compared between the novel strains, their closely related congeners, and *Deinococcus radiodurans* NBRC 15346ᵀ. This analysis aimed to investigate DNA repair-related mechanisms potentially contributing to the UV resistance of the strains.

In bacteria, the UvrABC system is pivotal in repairing DNA damage caused by UV radiation [48]. The UvrABC endonuclease complex operates in nucleotide excision repair (NER), a pathway critical for maintaining genomic stability. UvrA detects DNA damage, UvrB unwinds the DNA and UvrC cleaves the damaged regions. Additionally, the UvrABC complex interacts with RecA, a protein essential for DNA recombination and repair, which binds to damaged DNA and facilitates strand exchange with a new DNA strand. RecA is crucial for genome stability and enhanced UV resistance.

*Deinococcus radiodurans* is widely recognized for its exceptional radiation resistance and ability to withstand high levels of ionizing radiation. The functionality of its RecA system has been extensively documented [49,50], demonstrating its essential role in preserving genome integrity and conferring UV resistance.

*Pontibacter* species have also been reported to exhibit UV resistance [10,15, 16]. This study identified three novel *Pontibacter* strains from UV-irradiated soil, and their potential UV resistance was evaluated. The whole genome sequences of these three strains, along with their closely related congeners and *Deinococcus radiodurans*, were annotated using the RAST server. The annotated data identified protein-coding genes related to the UvrABC and RecA subsystems. Using BLAST_P_, the functional protein sequences were compared to assess similarities across the strains. This analysis provided insights into these proteins' amino acid sequences and genomic contexts, shedding light on their roles in DNA repair. The similarity percentages of Excinuclease ABC subunits (UvrA, UvrB and UvrC) and RecA across the studied strains and related species are detailed in Table S5.

### UV radiation resistance

**Fig. 3.**
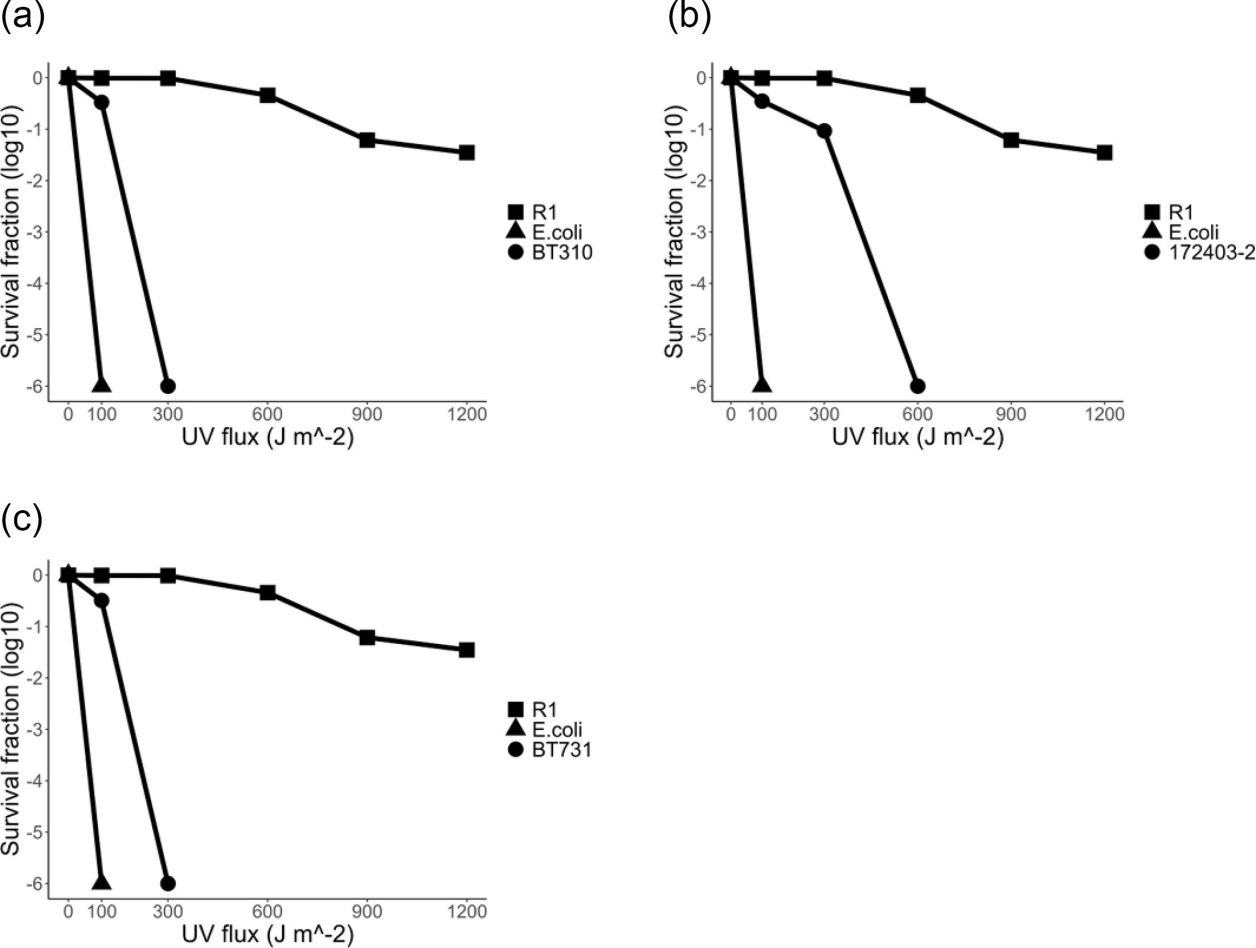
UV resistance profiles of the strains isolated in this study: 172403-2^T^ (**a**), BT310^T^ (**b**) and BT731^T^ (**c**). The survival rates of *Deinococcus radiodurans* R1^T^ (■; positive control), *Escherichia coli* K12 (▲; negative control) and strains (●) are shown.

In the UV resistance experiment, strain 172403-2^T^ exhibited moderate resistance, with survival gradually decreasing as UV flux increased. Conversely, strains BT310^T^ and BT731^T^ exhibited a rapid decline in survival at low UV flux levels, indicating a lack of resistance. Figure 3 illustrates the results of the UV resistance test.

### Chemotaxonomic characterization

The chemotaxonomic analyses were performed to determine the cellular fatty acid compositions of strains 172403-2^T^, BT310^T^ and BT731^T^, as well as those of their closest phylogenetic relatives. The dominant fatty acids in strain 172403-2^T^ were iso-C_15:0_ (28.7%) and Summed Feature 4 (iso-C_17:1_ I/anteiso-C_17:1_ B; 22.1%) (Table 3). Similarly, those of strain BT310^T^ were iso-C_15:0_ (29.5%) and Summed Feature 4 (20.9%), while for strain BT731^T^, they again were iso-C_15:0_ (16.3%) and Summed Feature 4, but Summed Feature 4 represented a significantly higher proportion (41.5%) (Table 4).

**Table 3.** Cellular fatty acid profiles of strain 172403-2^T^ and closely related species (tr, trace [< 1%]; nd, not detected). Numbers denote the strains: 1, 172403-2^T^; 2, *Pontibacter chitinilyticus* 17 gy-14^T^; 3, *Pontibacter arcticus* 2b14^T^; 4, *Pontibacter liquoris* NBU2971^T^. The data for strain 172403-2^T^ and the reference strains were obtained in this study unless otherwise indicated

Fatty acid	1	2	3	4
**Saturated**				
C_16:0_	tr	tr	3.6	tr
C_17:0_	1.2	1.5	1.2	nd
**Unsaturated**				
C_14:1_ *ω5*c	nd	5.0	nd	nd
C_16:1_ *ω5*c	4.3	2.2	6.4	4.8
C_17:1_ *ω6*c	6.3	2.1	2.2	1.1
**Branched-chain fatty acid**				
C_15:0_ iso	28.7	20.9	21.1	39.5
C_15:0_ iso 3OH	3.1	2.5	3.7	1.4
C_15:1_ iso F	nd	1.8	nd	1.8
C_16:0_ iso	2.4	nd	4.3	2.8
C_16:1_ iso H	2.0	2.6	1.8	1.8
C_17:0_ iso	5.6	4.5	7.3	1.4
C_17:0_ iso 3OH	9.6	8.5	14.1	5.4
C_18:1_ iso H	nd	nd	1.1	TR
C_15:0_ anteiso	4.5	11.1	nd	8.9
C_17:0_ anteiso	2.3	3.6	nd	1.1
**Summed feature**				
**3**; C_16:1_ *ω7*c/C1_6:1_ *ω6*c	2.2	5.3	3.1	2.0
**4**; C_17:1_ iso I/C_17:1_ anteiso B	22.1	26.2	22.2	13.8
**9**; C_17:1_ iso *ω*9c/C_16:0_ 10-methyl	nd	nd [3]	tr [8]	5.6 [51]

**Table 4.** Cellular fatty acid profiles of strains BT310^T^ and BT731^T^ and closely related species (TR, trace [< 1%]; nd, not detected). Numbers denote the strains: 1, BT310^T^; 2, BT731^T^; 3, *Pontibacter pudoricolor* BT214^T^; 4, *Pontibacter populi* HYL7-15^T^; 5, *Pontibacter virosus* W14^T^; and 6, *Pontibacter amylolyticus* 9-2^T^. The data of all strains were obtained in this study

Fatty acid	1	2	3	4	5	6
**Saturated**						
C_16:0_	tr	tr	2.4	tr	tr	1.1
C_17:0_	tr	tr	1.3	tr	tr	1.0
**Unsaturated**						
C_15:1_ *ω6*c	1.7	tr	1.9	1.2	tr	1.0
C_16:1_ *ω5*c	6.3	4.7	2.7	3.6	3.8	3.3
C_17:1_ *ω6*c	6.2	1.8	11.0	4.3	2.8	11.6
C_18:1_ *ω9*c	nd	1.8	nd	1.8	1.4	nd
**Branched-chain fatty acid**						
C_15:0_ iso	29.5	16.3	30.3	29.6	20.8	22.0
C_15:0_ iso 3OH	5.5	2.1	3.4	4.3	4.1	6.3
C_15:1_ iso G	1.2	2.4	nd	1.1	TR	nd
C_16:0_ iso	nd	tr	3.4	1.2	2.0	2.7
C_16:1_ iso H	4.5	2.4	3.2	1.6	1.4	1.8
C_17:0_ iso	tr	3.5	tr	1.5	1.6	1.2
C_17:0_ iso 3OH	6.8	6.9	7.0	9.6	9.5	10.2
C_15:0_ anteiso	2.4	5.4	1.6	1.0	5.4	2.7
C_17:0_ anteiso	nd	3.1	nd	2.2	2.4	tr
**Summed feature**						
**3**; C_16:1_ *ω7*c/C1_6:1_ *ω6*c	1.6	tr	tr	1.6	tr	tr
**4**; C_17:1_ iso I/C_17:1_ anteiso B	3.6	1.9	3.1	2.0	1.7	2.6
**9**; C_17:1_ iso *ω*9c/C_16:0_ 10-methyl	20.9	41.5	21.5	30.3	37.4	26.4

The major polar lipid identified in all three strains was PE. The polar lipid profile of strain 172403-2^T^ included PE, along with one amino lipid, one aminophospholipid, two glycolipids and two unidentified polar lipids (Fig. S7). Similarly, the polar lipid composition of strain BT310^T^ consisted of PE, one amino lipid, one aminophospholipid, one glycolipid, two phospholipids and seven unidentified polar lipids (Fig. S8), and that of strain BT731^T^ contained PE, two phospholipids, two glycolipids, one aminophospholipid and three unidentified polar lipids (Fig. S9). All three strains exhibited MK-7 as the predominant respiratory quinone, a characteristic commonly found within the genus *Pontibacter*.

Considering the polyphasic results presented, we considered strains 172403-2^T^, BT310^T^ and BT731^T^ to represent three novel species within the genus *Pontibacter*, for which the names *Pontibacter rufus* nov. sp., *Pontibacter humidus* nov. sp. and *Pontibacter coccineus* nov. sp. are proposed. The 16S rRNA sequences of strains 172403-2^T^, BT310^T^ and BT731^T^ have been deposited in the NCBI database under the accession numbers MW237669, MT795756 and OP795871, corresponding to each strain, respectively. These findings contribute to the expanding knowledge of microbial diversity within the genus *Pontibacter*.

## Description of *Pontibacter rufus* sp. nov.

*Pontibacter rufus* (ru'fus. L. masc. adj. *rufus*, red, reddish).

The cells are Gram negative, non-flagellated, non-motile and short rod shaped. On R2A agar, colonies are circular, convex, mucoid and exhibit a red colour after 72 h of incubation at 30 °C. Cell dimensions range from approximately 1.0 to 1.5 µm in width and 2.0 to 2.7 µm in length. Growth of strain 172403-2^T^ occurs within a temperature range of 10–37°C and at NaCl concentrations of 0.0%–4.0% (w/v) and pH levels of 6.0–8.0, with optimal growth at 30 °C, 1.5% NaCl and pH 7.0. The strain grows on R2A agar, TSA, LB and NA but does not thrive on MAC agar. Both oxidase and catalase activities are positive.

In strain 172403-2^T^, the primary respiratory quinone is MK-7. The predominant cellular fatty acids are iso-C_15:0_ and summed feature 4 (comprising iso-C_17:1_ I and anteiso-C_17:1_ B). The major polar lipid identified is PE.

Strain 172403-2^T^ (= KCTC 62072^T^ = NBRC 114967^T^), the type strain for *Pontibacter rufus*, was first isolated from soil in Korea. The genome sequence of strain 172403-2^T^ has been deposited and is accessible in the GenBank, DDBJ and EMBL databases under the accession number JADQDR000000000. The 16S rRNA gene sequence of the strain is registered and searchable in GenBank under the accession number MW237669.

## Description of *Pontibacter humidus* sp. nov.

*Pontibacter humidus* (hu'mi.dus. L. masc. adj. *humidus*, moist, humid, wet).

The cells are Gram negative, non-flagellated, non-motile and short rod shaped. On R2A agar, the colonies are circular, convex, moist and exhibit a light pink colour after 72 h of incubation at 30 °C. The cell dimensions are approximately 0.9 to 1.1 µm in width and 2.2 to 2.4 µm in length. Strain BT310^T^ grows within a temperature range of 20–37 °C, tolerates 0.0–4.0% (w/v) NaCl and grows at pH levels of 6.0–8.0, with optimal growth at 30 °C, 1.5% NaCl and pH 7.0. This strain grows on R2A agar, TSA, LB and NA but not on MAC agar. Both oxidase and catalase tests are positive.

Strain BT310^T^ contains MK-7 as its primary respiratory quinone. The dominant cellular fatty acids are Summed Feature 4 (comprising iso-C_17:1_ I and anteiso-C_17:1_ B) and iso-C_15:0_. Phosphatidylethanolamine is the major polar lipid detected.

The type strain for *Pontibacter humidus*, designated BT310^T^ (= KCTC 72363^T^ = NBRC 114846^T^), was first isolated from soil in Korea. The genome sequence of strain BT310^T^ has been deposited and is accessible in the GenBank, DDBJ and EMBL databases under the accession number JAELXU000000000. The 16S rRNA gene sequence of the strain is registered and searchable in GenBank under the accession number MT795756.

## Description of *Pontibacter coccineus* sp. nov.

*Pontibacter coccineus* (coc.ci’ne.us. L. masc. adj. *coccineus*, reddish coloured).

The cells are Gram negative, non-flagellated, non-motile and rod shaped. On R2A agar, the colonies are circular, convex, smooth and exhibit an orange-red colour after 72 h of incubation at 30 °C. The cell dimensions range from approximately 0.7 to 1.2 µm in width and 1.8 to 2.6 µm in length. Strain BT731^T^ grows within a temperature range of 10–35 °C, tolerates NaCl concentrations from 0.0% to 3.5% (w/v) and a pH range of 6.0–8.0, with optimal growth at 30 °C, no NaCl and pH 7.0. This strain grows on R2A agar, TSA, LB and NA but not MAC agar. Oxidase activity is positive, while catalase activity is negative.

The primary respiratory quinone in strain BT731^T^ is MK-7. The major cellular fatty acids are iso-C_15:0_ and Summed Feature 4 (comprising iso-C_17:1_ I and anteiso-C_17:1_ B). Phosphatidylethanolamine is the major polar lipid detected.

The type strain for *Pontibacter coccineus*, designated BT731^T^ (= KCTC 92910^T^ = NBRC 116069^T^), was first isolated from soil in Korea. The genome sequence of strain BT731^T^ has been deposited and is accessible in the GenBank, DDBJ and EMBL databases under the accession number JAUOTN000000000. The 16S rRNA gene sequence of the strain is registered and searchable in GenBank under the accession number OP795871.

## Supplementary material

10.1099/ijsem.0.006755 Uncited Supplementary Material 1.

## Abbreviations

ANI: average nucleotide identity
APL: aminophospholipid
BLAST: Basic Local Alignment Search Tool
CDSs: coding sequences
dDDH: digital DNA–DNA hybridization
GGDC: Genome-to-Genome Distance Calculator
GTDB: Genome Taxonomy Database
iTOL: Interactive Tree Of Life
LB: lysogeny broth
MCA: MacConkey agar
ML: maximum likelihood
MP: maximum parsimony
NA: nutrient agar
NCBI: National Center for Biotechnology Information
NJ: neighbour joining
PE: phosphatidylethanolamine
PL: phospholipids
R2A: Reasoner’s 2A
RAST: Rapid Annotation using Subsystem Technology
TSA: tryptic soy agar
UBCG: Up-to-date Bacterial Core Gene
