# Supplementary material for: Pontibacter rufus sp. nov., Pontibacter humidus sp. nov. and Pontibacter coccineus sp. nov. isolated from UV-irradiated soil in Korea
Source: Int J Syst Evol Microbiol. 2025 Apr 28;75(4):006755. doi: 10.1099/ijsem.0.006755 (PMC12281811; doi:10.1099/ijsem.0.006755)

Supplementary Material

Pontibacter rufus sp. nov., *Pontibacter humidus* sp. nov., and *Pontibacter coccineus* sp. nov., isolated from UV-irradiated soil in Korea

Seona Park[1], Hyang Burm Lee[2]*, Sathiyaraj Srinivasan[1]*, and Myung Kyum Kim[1]*

[1]Department of Bio & Environmental Technology, College of Natural Science, Seoul Women's University, Seoul 01797, Korea

[2]Environmental Microbiology Lab, Department of Agricultural Biological Chemistry, College of Agriculture & Life Sciences, Chonnam National University, Gwangju 61186, South Korea

*To whom correspondence should be addressed:

: Myung Kyum Kim. Tel: +82-2-970-5667. E-mail: biotech@swu.ac.kr

: Sathiyaraj Srinivasan. Tel: +82-2-970-5670. E-mail: drsrini@swu.ac.kr

: Hyang Burm Lee. Tel: +82-62-530-2136. E-mail: hblee@chonnam.ac.kr

Contents

5 supplementary tables

9 supplementary figures

Table S1. Genome assembly statistics of strain 172403-2^T, BT310^T, BT731^T, and the closely related *Pontibacter* species.

Data on the related type strains were retrieved from GenBank (https://www.ncbi.nlm.nih.gov/genbank/).

Taxa: 1, strain 172403-2^T; 2, strain BT310^T; 3, strain BT731^T; 4, *P. chitinilyticus* 17gy-14^T; 5, *P. arcticus* 2b14^T; 6, *P. liquoris* NBU2971^T; 7, *P. pudoricolor* BT214^T; 8, *P. populi* HYL7-15^T; 9, *P. virosus* W14^T; and 10, *P. amylolyticus* 9-2^T.

	1	2	3	4	5	6	7	8	9	10
Genome size (bp)	5,076,851	4,294,440	4,655,665	4,854,262	3,773,432	4,917,612	4,344,619	4,363,441	4,884,877	4,588,841
Coverage (X)	29.7	45.8	38.3	43.7	503.0	100.0	201.5	73.8	229.0	105.0
Number of contigs	41	9	12	24	7	4	1	31	47	16
N50 (bp)	363,827	1,220,683	627,395	274,139	1,004,009	3,217,459	4,344,619	236,506	282,824	209,152
L50	5	2	4	5	2	1	1	7	6	6
Protein coding genes (CDSs)	4,951	3,901	4,347	4,538	3,512	4,599	4,095	4,036	4,539	4,239
tRNA genes	38	38	41	38	45	41	45	39	42	44
G+C content (%)	48.6	45.2	51.3	50.5	45.5	51.3	45.4	45.0	50.1	51.8

Table S2. Whole genome-based average nucleotide identity (ANI) and digital DNA–DNA hybridization (DDH) values between strain 172403-2^T and the type strains of *Pontibacter* species with published genomes. Data for related type strains were retrieved from NCBI (https://www.ncbi.nlm.nih.gov/).

			Strain 172403-2	
**Species**	**Strain**	**Genome**	**ANI (%)**	**DDH (%)**
Pontibacter liquoris	NBU2971^T	GCA 022758235	76.87	19.93
Pontibacter chitinilyticus	17gy-14^T	GCA 039748935	76.37	20.63
Pontibacter actiniarum	DSM 19842^T	GCA 003585765	74.45	21.20
Pontibacter russatus	BT326^T	GCA 009931655	74.42	21.59
Pontibacter mangrovi	HB172049^T	GCA 006385705	74.07	22.21
Pontibacter flavimaris	S10-8^T	GCA 001907195	73.96	22.17
Pontibacter akesuensis	AKS 1^T	GCA 001611675	73.81	22.42
Pontibacter ruber	JC213^T	GCA 023630205	73.71	22.87
Pontibacter chinhatensis	LP51^T	GCA 900113285	73.63	22.18
Pontibacter mucosus	DSM 100162^T	GCA 003054055	73.61	22.35
Pontibacter diazotrophicus	H4X^T	GCA 003367245	73.57	22.67
Pontibacter pamirensis	TRT317^T	GCA 010015475	73.39	22.26
Pontibacter oryzae	KIRAN^T	GCA 003576375	73.15	22.53
Pontibacter arcticus	2b14^T	GCA 003284895	73.06	22.51
Pontibacter roseus	DSM 17521^T	GCA 000373265	72.96	22.78
Pontibacter ummariensis	NKM1^T	GCA 900188175	72.96	22.50
Pontibacter vulgaris	NBU2972^T	GCA 022758145	72.81	23.06
Pontibacter amylolyticus	CGMCC 1.12749^T	GCA 014638845	72.78	22.85
Pontibacter cellulosilyticus	SD6^T	GCA 014306105	72.74	23.58
Pontibacter aquaedesilientis	JH31^T	GCA 014773225	72.63	23.16
Pontibacter ramchanderi	LP43^T	GCA 002846395	72.61	23.50
Pontibacter indicus	LP100^T	GCA 900156345	72.56	23.18
Pontibacter pudoricolor	BT214^T	GCA 010092985	72.46	22.75
Pontibacter virosus	DSM 100231^T	GCA 003096355	72.42	22.94
Pontibacter korlensis	X14-1^T	GCA 000973725	72.40	23.04
Pontibacter lucknowensis	DM9^T	GCA 900156415	72.35	23.47
Pontibacter deserti	JC215^T	GCA 023630255	72.22	23.39
Pontibacter populi	HLY7-15^T	GCA 040278685	72.15	23.66
Pontibacter harenae	XAAS-A31^T	GCA 020907245	71.83	23.24
Pontibacter silvestris	XAAS-R86^T	GCA 020907275	71.70	22.94

Table S3. Whole genome-based average nucleotide identity (ANI) and digital DNA–DNA hybridization (DDH) values between strain BT310^T and the type strains of *Pontibacter* species with published genomes. Data for related type strains were retrieved from NCBI (https://www.ncbi.nlm.nih.gov/).

			Strain BT310^T	
**Species**	**Strain**	**Genome**	**ANI (%)**	**DDH (%)**
Pontibacter pudoricolor	BT214^T	GCA 010092985	88.56	11.55
Pontibacter populi	HLY7-15^T	GCA 040278685	83.46	15.82
Pontibacter deserti	JC215^T	GCA 023630255	78.81	19.49
Pontibacter vulgaris	NBU2972^T	GCA 022758145	74.21	21.51
Pontibacter ruber	JC213^T	GCA 023630205	73.33	22.30
Pontibacter arcticus	2b14^T	GCA 003284895	72.98	22.90
Pontibacter cellulosilyticus	SD6^T	GCA 014306105	72.77	22.21
Pontibacter flavimaris	S10-8^T	GCA 001907195	72.74	22.38
Pontibacter liquoris	NBU2971^T	GCA 022758235	72.50	22.37
Pontibacter mucosus	DSM 100162^T	GCA 003054055	72.38	22.80
Pontibacter mangrovi	HB172049^T	GCA 006385705	72.33	23.58
Pontibacter chinhatensis	LP51^T	GCA 900113285	72.30	23.04
Pontibacter amylolyticus	CGMCC 1.12749^T	GCA 014638845	72.28	22.94
Pontibacter indicus	LP100^T	GCA 900156345	72.14	22.98
Pontibacter ramchanderi	LP43^T	GCA 002846395	72.13	22.94
Pontibacter actiniarum	DSM 19842^T	GCA 003585765	72.09	23.15
Pontibacter virosus	DSM 100231^T	GCA 003096355	72.05	23.17
Pontibacter akesuensis	AKS 1^T	GCA 001611675	71.97	23.36
Pontibacter aquaedesilientis	JH31^T	GCA 014773225	71.94	23.70
Pontibacter lucknowensis	DM9^T	GCA 900156415	71.93	23.14
Pontibacter roseus	DSM 17521^T	GCA 000373265	71.88	23.56
Pontibacter oryzae	KIRAN^T	GCA 003576375	71.84	23.69
Pontibacter pamirensis	TRT317^T	GCA 010015475	71.83	23.28
Pontibacter diazotrophicus	H4X^T	GCA 003367245	71.80	23.12
Pontibacter korlensis	X14-1^T	GCA 000973725	71.76	23.11
Pontibacter chitinilyticus	17gy-14^T	GCA 039748935	71.61	23.73
Pontibacter russatus	BT326^T	GCA 009931655	71.58	24.66
Pontibacter harenae	XAAS-A31^T	GCA 020907245	71.55	23.21
Pontibacter ummariensis	NKM1^T	GCA 900188175	71.23	23.63
Pontibacter silvestris	XAAS-R86^T	GCA 020907275	71.17	23.62

Table S4. Whole genome-based average nucleotide identity (ANI) and digital DNA–DNA hybridization (DDH) values between strains BT731^T and the type strains of *Pontibacter* species with published genomes. Data for related type strains were retrieved from NCBI (https://www.ncbi.nlm.nih.gov/).

			Strain BT731^T	
**Species**	**Strain**	**Genome**	**ANI (%)**	**DDH (%)**
Pontibacter amylolyticus	CGMCC 1.12749^T	GCA 014638845	87.66	12.21
Pontibacter virosus	DSM 100231^T	GCA 003096355	85.45	14.14
Pontibacter indicus	LP100^T	GCA 900156345	84.26	15.36
Pontibacter ramchanderi	LP43^T	GCA 002846395	82.85	16.48
Pontibacter lucknowensis	DM9^T	GCA 900156415	82.64	16.55
Pontibacter aquaedesilientis	JH31^T	GCA 014773225	78.29	19.51
Pontibacter roseus	DSM 17521^T	GCA 000373265	77.38	20.13
Pontibacter ruber	JC213^T	GCA 023630205	74.39	21.71
Pontibacter flavimaris	S10-8^T	GCA 001907195	73.94	21.76
Pontibacter mucosus	DSM 100162^T	GCA 003054055	73.57	21.74
Pontibacter chinhatensis	LP51^T	GCA 900113285	73.52	21.93
Pontibacter russatus	BT326^T	GCA 009931655	73.33	22.28
Pontibacter mangrovi	HB172049^T	GCA 006385705	73.29	22.11
Pontibacter actiniarum	DSM 19842^T	GCA 003585765	73.10	22.44
Pontibacter liquoris	NBU2971^T	GCA 022758235	73.06	22.38
Pontibacter akesuensis	AKS 1^T	GCA 001611675	72.79	22.54
Pontibacter chitinilyticus	17gy-14^T	GCA 039748935	72.34	23.09
Pontibacter vulgaris	NBU2972^T	GCA 022758145	72.25	23.19
Pontibacter pudoricolor	BT214^T	GCA 010092985	72.22	22.55
Pontibacter ummariensis	NKM1^T	GCA 900188175	72.17	22.51
Pontibacter diazotrophicus	H4X^T	GCA 003367245	72.09	22.72
Pontibacter populi	HLY7-15^T	GCA 040278685	72.04	23.20
Pontibacter pamirensis	TRT317^T	GCA 010015475	72.00	22.89
Pontibacter oryzae	KIRAN^T	GCA 003576375	71.93	23.40
Pontibacter cellulosilyticus	SD6^T	GCA 014306105	71.84	23.04
Pontibacter arcticus	2b14^T	GCA 003284895	71.81	23.55
Pontibacter deserti	JC215^T	GCA 023630255	71.75	23.08
Pontibacter korlensis	X14-1^T	GCA 000973725	71.65	22.73
Pontibacter harenae	XAAS-A31^T	GCA 020907245	71.26	22.72
Pontibacter silvestris	XAAS-R86^T	GCA 020907275	70.41	24.13

Table S5. Similarity (%; based on BLASTp) of DNA repair genes involved in the excision repair pathways of the three novel strains and related *Pontibacter* strains compared to *Deinococcus radiodurans* NBRC 15346^T (www.ncbi.nlm.nih.gov/). Numbers denote the strains: 1, 172403-2^T; 2, BT310^T; 3, BT731^T; 4, *P. chitinilyticus* 17gy-14^T; 5, *P. arcticus* 2b14^T; 6, *P. liquoris* NBU2971^T; 7, *P. pudoricolor* BT214^T; 8, *P. populi* HYL7-15^T; 9, *P. virosus* W14^T; and 10, *P. amylolyticus* 9-2^T.

**Gene**	**Amino acid similarity (%)**									
	**1**	**2**	**3**	**4**	**5**	**6**	**7**	**8**	**9**	**10**
Excinuclease ABC subunit UvrA	51.70	51.29	50.88	52.21	51.08	51.18	51.08	51.29	50.98	50.98
Excinuclease ABC subunit UvrB	51.94	52.61	52.91	51.86	52.16	52.76	52.46	53.40	52.31	52.76
Excinuclease ABC subunit UvrC	30.31	30.08	29.24	30.15	29.10	30.58	30.24	30.25	29.41	29.42
RecA protein	55.66	55.66	55.05	56.21	55.66	56.27	55.66	55.66	55.05	55.05

Fig. S1. Maximum likelihood phylogenetic tree based on 16S rRNA gene sequences showing the positions of strains 172403-2^{T}, BT310^{T}, and BT731^{T} among other species of the genus *Pontibacter*. The numbers at the nodes are bootstrap percentages (> 70%). *Hymenobacter roseosalivarius* AA-718^{T} was used as the outgroup. The bar represents 0.01 substitutions per nucleotide position.

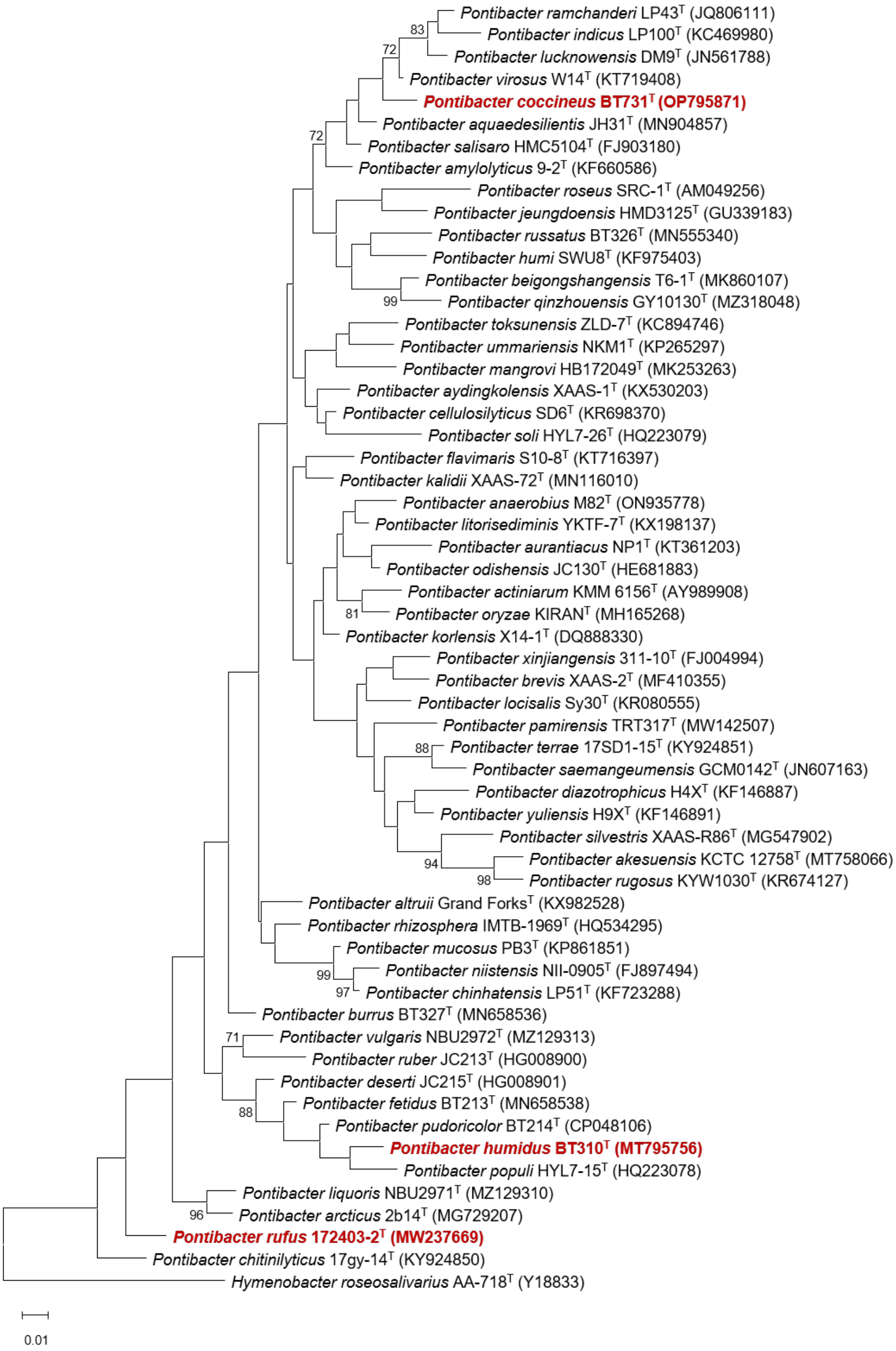

Fig. S2. Maximum parsimony phylogenetic tree based on 16S rRNA gene sequences showing the positions of strains 172403-2^T, BT310^T, and BT731T^T among other species of the genus *Pontibacter*. The numbers at the nodes are bootstrap percentages (> 70%). *Hymenobacter roseosalivarius* AA-718^T was used as the outgroup.

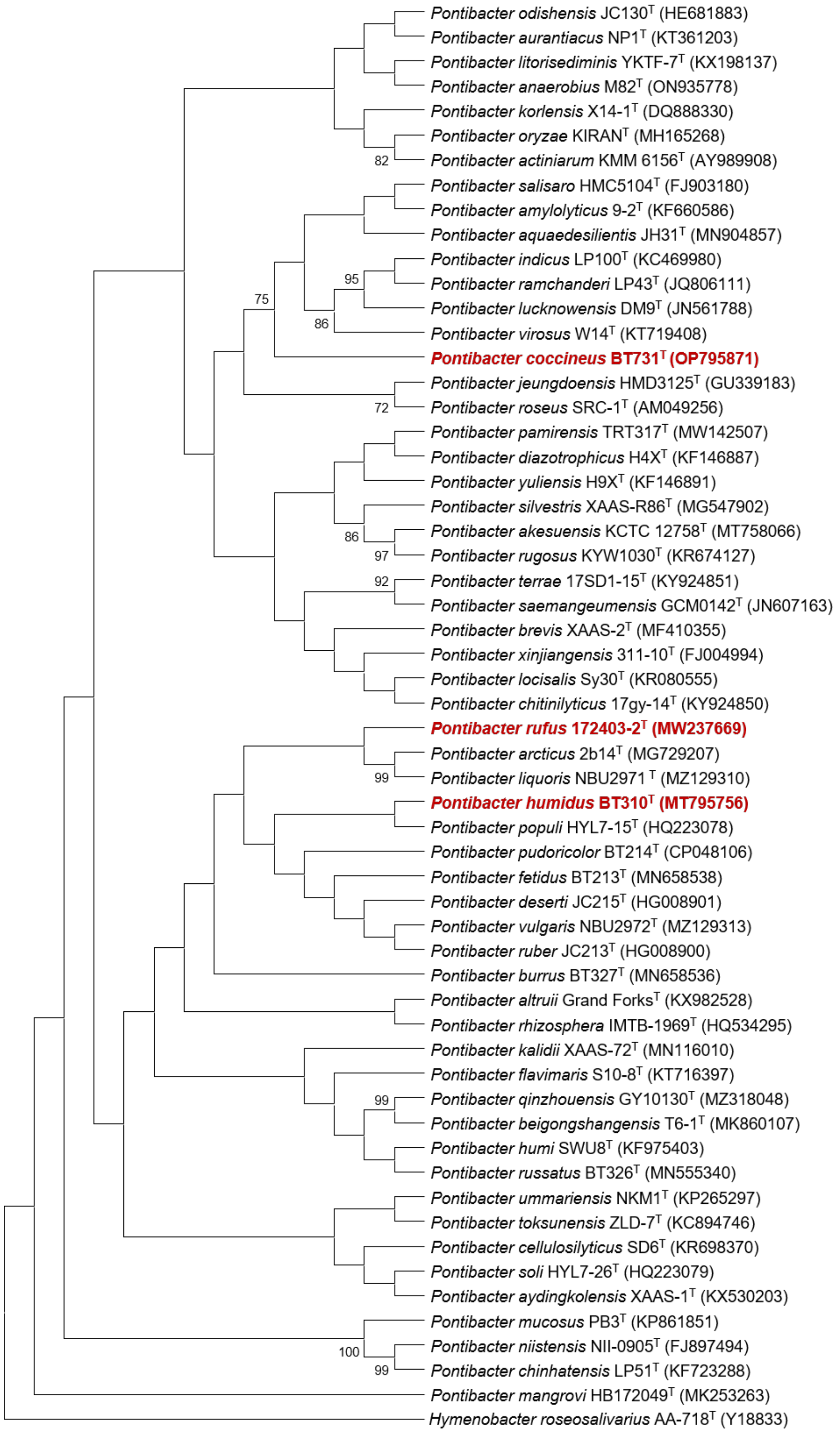

Fig. S3. UBCG phylogenetic tree constructed from a comparative analysis of whole genome sequences showing the relationships of strains 172403-2^{T}, BT310^{T}, and BT731T^{T} among other species of the genus *Pontibacter* with validly published genomes. *Hymenobacter roseosalivarius* AA-718^{T} was used as the outgroup. The bar represents 0.10 substitutions per position.

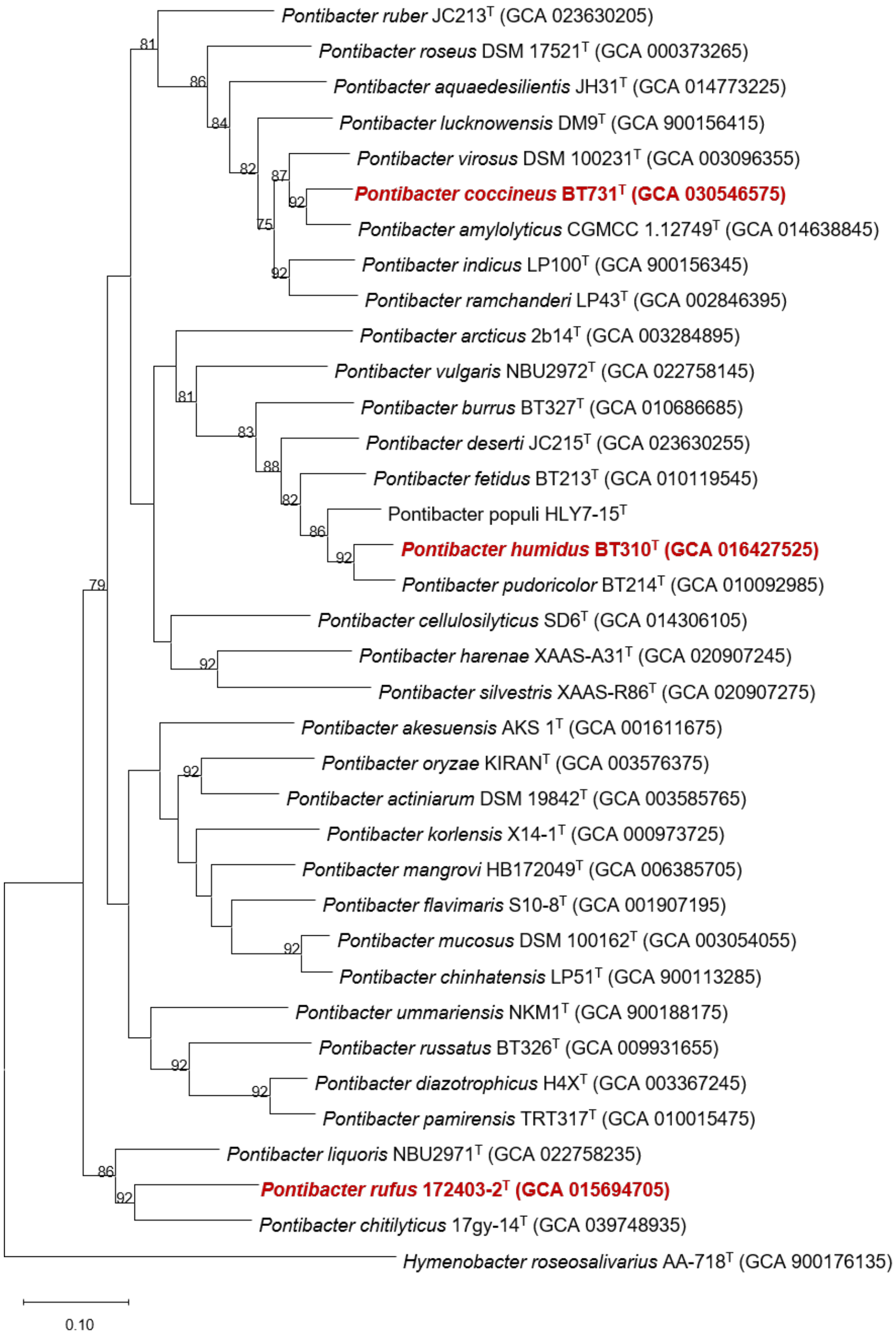

Fig. S4. The subsystem category distribution of genes in strain 172403-2^T, expressed as percentages, as determined using the RAST annotation server.

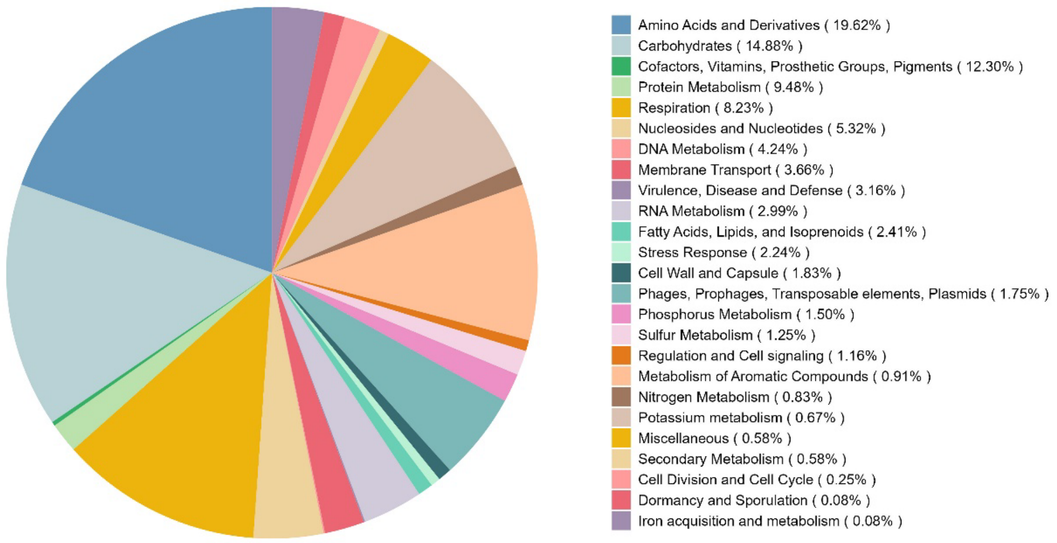

Fig. S5. The subsystem category distribution of genes in strain BT310^{T}, expressed as percentages, as determined using the RAST annotation server.

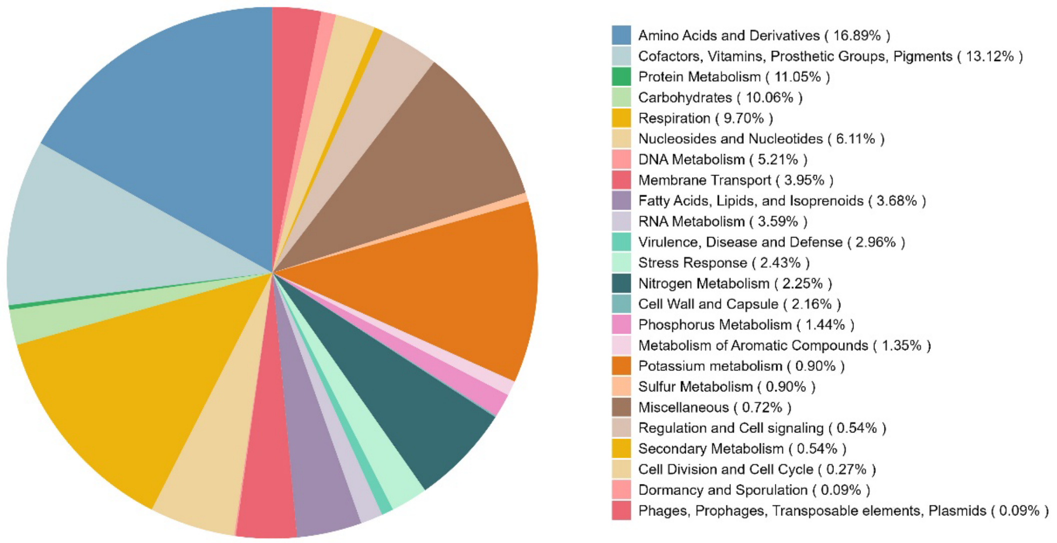

Fig. S6. The subsystem category distribution of genes in strain BT731^T, expressed as percentages, as determined using the RAST annotation server.

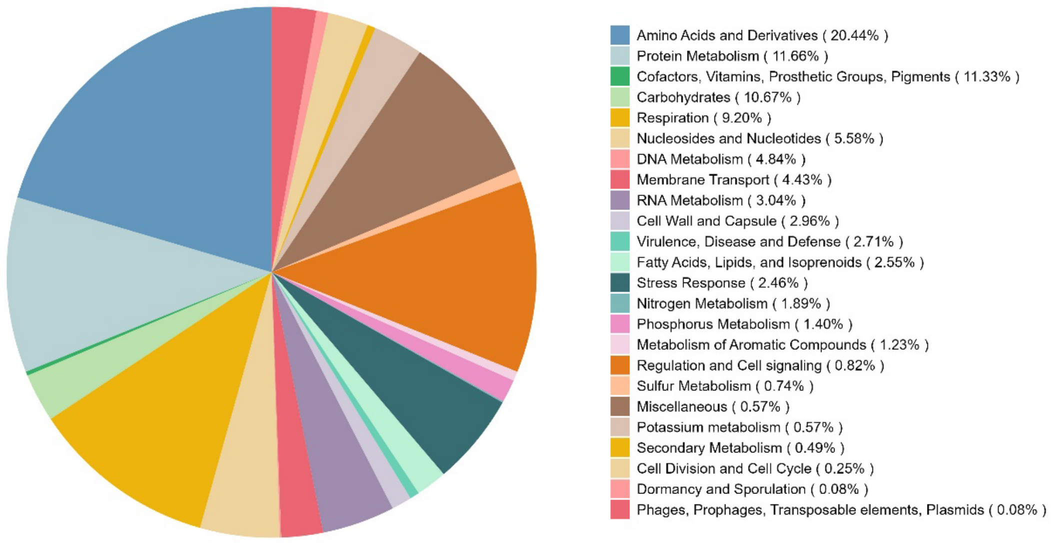

Fig. S7. Polar lipid profiles of strain 172403-2^T after two-dimensional chromatography for total lipids (a), phospholipids (b), aminolipids (c), and glycolipids (d). The abbreviations include L, lipid; PL, phospholipid; APL, aminophospholipid; PE, phosphatidylethanolamine; AL, aminolipid; and GL, glycolipid.

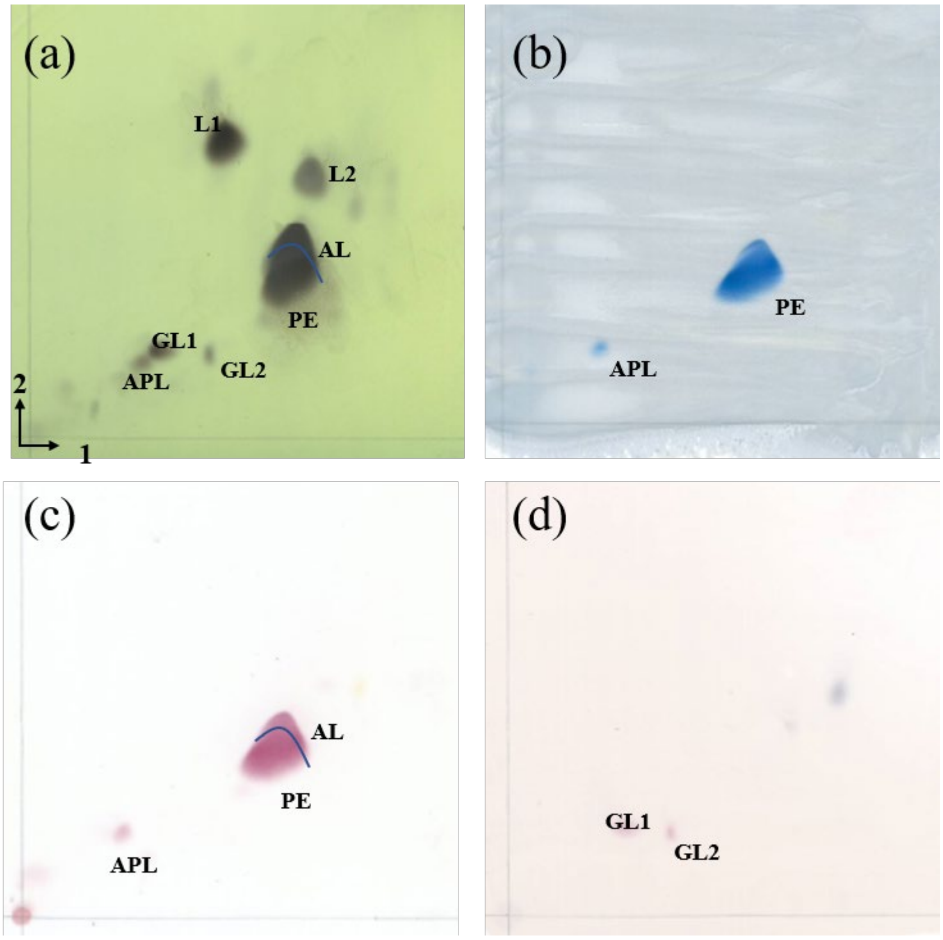

Fig. S8. Polar lipid profiles of strain BT310^T after two-dimensional chromatography for total lipids (a), phospholipids (b), aminolipids (c), and glycolipids (d). The abbreviations include L, lipid; PL, phospholipid, APL, aminophospholipid; PE, phosphatidylethanolamine; AL, aminolipid; and GL, glycolipid.

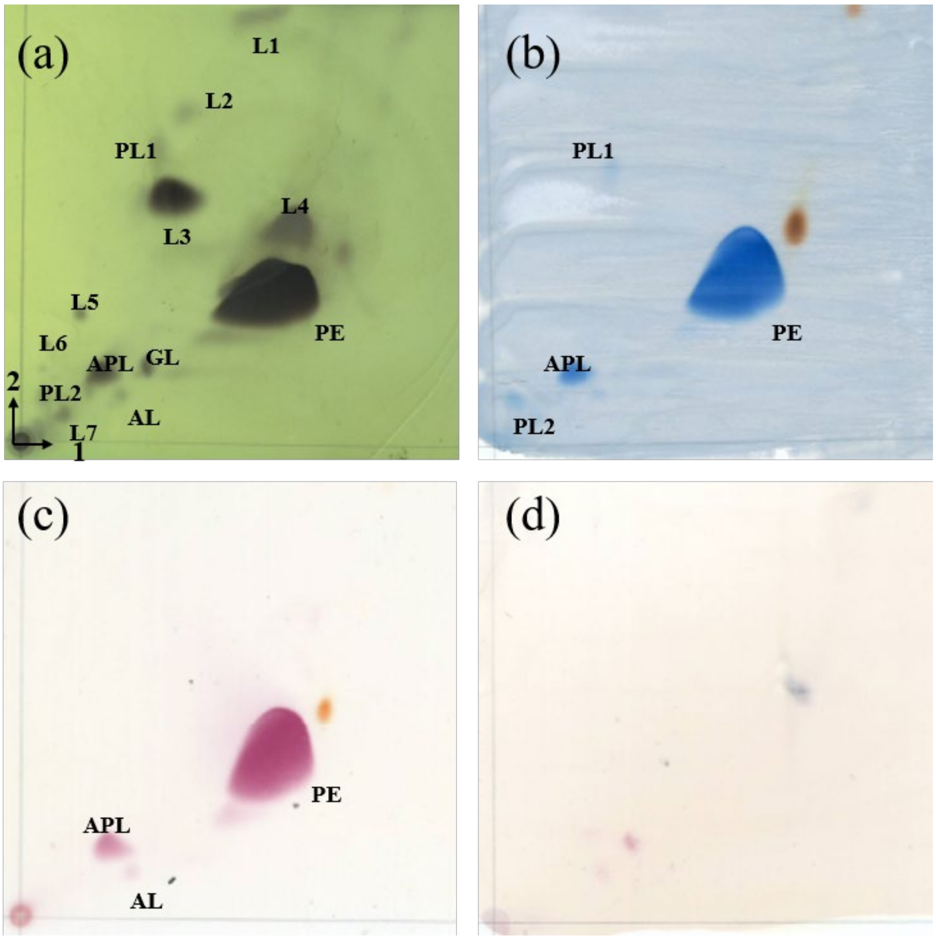

Fig. S9. Polar lipid profiles of strain BT731^T after two-dimensional chromatography for total lipids (a), phospholipids (b), aminolipids (c), and glycolipids (d). The abbreviations include L, lipid; PL, phospholipid; APL, aminophospholipid; PE, phosphatidylethanolamine; and GL, glycolipid

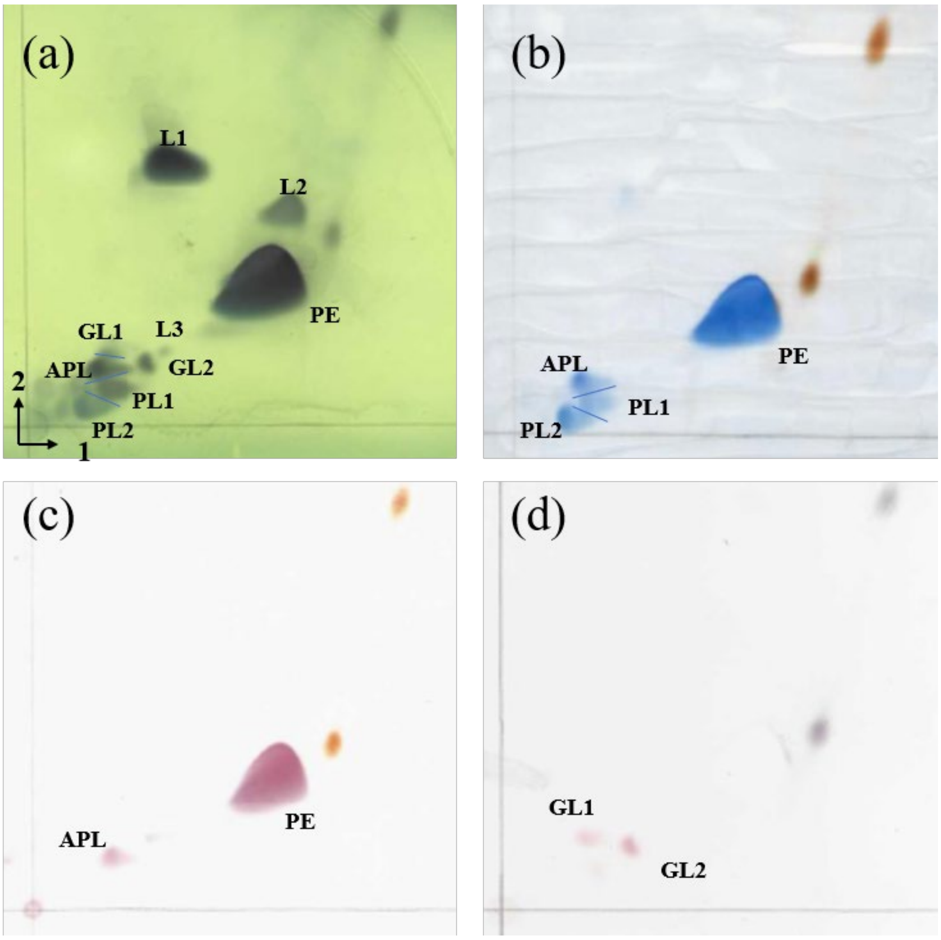

Supplement: Uncited Supplementary Material 1. [file ijsem-75-06755-s001.pdf]
